# Spinal Syphilitic Gumma: A Rare Presentation of an Old Disease

**DOI:** 10.1155/2021/5533686

**Published:** 2021-06-02

**Authors:** Dominic Worku, Angela Houston, Catherine Cosgrove, Laura Byrne

**Affiliations:** Clinical Infection Unit, St. George's Hospital, BlackshawRoad, Tooting, London SW17 0QT, UK

## Abstract

Syphilis is an ancient condition which still is of global concern today. Despite better awareness amongst clinicians and improving diagnostics, it remains likely underdiagnosed in part because of its namesake the ‘great imitator.' While many patients suffer primary or secondary disease, tertiary syphilis characterised by gumma is rare, especially in the context of neurosyphilis. Here, we report a rare case of a well-controlled human immunodeficiency virus- (HIV-) positive gentleman with a history of previous syphilis and epilepsy who presented with progressive left leg weakness leading to immobility and altered bowels and, on neurological examination, Brown-Sequard syndrome. Magnetic resonance imaging (MRI) of the spine revealed two peripherally enhancing cavitating lesions at T4-T5 with associated meningeal thickening and cord oedema. Cerebrospinal fluid (CSF) analysis revealed high protein (3.07 g/dL) and white cell count (7 × 10^9^/L) with negative cryptococcal antigen, tuberculosis molecular testing (GeneXpert), microscopy and culture, and viral polymerase chain reaction (PCR). CSF serology was positive for *Treponema pallidum* particle agglutination (TPPA) 10240 and RPR 1 in 2 suggesting active disease. While TB treatment had been started prior to these investigations on day 11, 14-day high-dose benzylpenicillin therapy commenced. Repeat MRI of the spine at days 12 and 22 showed incremental improvements in all parameters which correlated with improving functionality and neurology. According to our literature search, this represents the 13^th^ case recorded for spinal syphilitic gumma and the only case recorded in a HIV-positive individual and adds to the evidence that, in the absence of rapidly changing neurology, medical management can lead to good clinical outcomes.

## 1. Introduction

Syphilis is an infection first recognised in the fifteenth century caused by the bacterial spirochete *Treponema pallidum* subsp. *pallidum* (*T. pallidum)* which can survive for decades in affected hosts and is heralded the ‘great imitator' for its varied and florid presentations [1, 2]. While cases of syphilis have dramatically declined since the advent of penicillin, there has, in recent years, been a resurgence with 5920 cases reported in England in 2016, the highest reported since 1949, and an 81% reported increase in syphilis infections in the USA between 2014 and 2018. As such, syphilis remains a major and global public health problem [1, 3].

Syphilis is predominantly acquired through sexual transmission and direct contact with an infectious lesion, with transmission rates varying between 10 and 60%. Vertical transmission is also possible although infrequent and remains an important component of antenatal screening in pregnancy. Important at-risk groups in whom syphilis majorly affects are men who have sex with men (MSM) and those with human immunodeficiency virus (HIV) which are reported in 80% and 40% of cases, respectively. The presence of syphilis is important for two reasons: firstly, syphilis can increase the risk of HIV transmission, and secondly, HIV can lead to suboptimal and incomplete responses of syphilis to treatment leading to more advanced disease [3, 4]. While different phases of syphilis infection are well recognised, patients may present with a significant overlap which can often complicate the diagnosis (Table 1); likewise, syphilis serology can often be difficult to interpret further leading to diagnostic delays. Increasingly, it has been recognised that *T. pallidum* disseminates early to the central nervous system (CNS), with asymptomatic or symptomatic neurologic involvement possible at any stage of the disease. However, of the 25–40% estimated to have neuroinvasion during the course of infection, predominantly, in the primary or secondary stage of the disease, the majority appear to resolve without receiving targeted CNS therapy. However, in the setting of concurrent HIV, more frequent, earlier, and severe CNS disease has been noted [3, 5]. Symptomatic neurological disease can be classified as being meningovascular or parenchymatous in nature and typically occur as part of tertiary syphilis around 5–15 years after primary infection [1, 6].

At present, neurosyphilis is a diagnosis in which available tests are suboptimal and are better at excluding the disease than confirming it. As such, careful interpretation of clinical signs, serum, and cerebrospinal fluid (CSF) findings is needed for a diagnosis to be made. Common findings include CSF pleocytosis, hyperproteinorrachia, oligoclonal bands, and increased IgG. While these are considered nonspecific markers of neurosyphilis, CSF serology in particular Venereal Disease Research Laboratory (VDRL) antibody is considered the gold standard; however, this remains an area of debate [6–8]. One rare presentation of tertiary syphilis is the formation of gumma which, historically, could be seen in up to 1 in 3 with untreated disease. The gumma is the characteristic lesion of tertiary syphilis, forms at sites of persistent infection, and is a form of granulomatous inflammation with a caseous core. Unlike tuberculomas, there are fewer lymphocytes, abundant plasma cells, and ghost cells present on histology, while the presence of necrosis and plasma cells essentially excludes sarcoidosis [9].

In this case report, we report a rare instance of spinal syphilitic gumma presenting as an incomplete Brown-Sequard syndrome. This represents the 13^th^ known case of spinal syphilitic gumma and is therefore a unique learning opportunity as to the varied behaviour of this common infection.

## 2. Clinical Presentation

**Figure 1:**
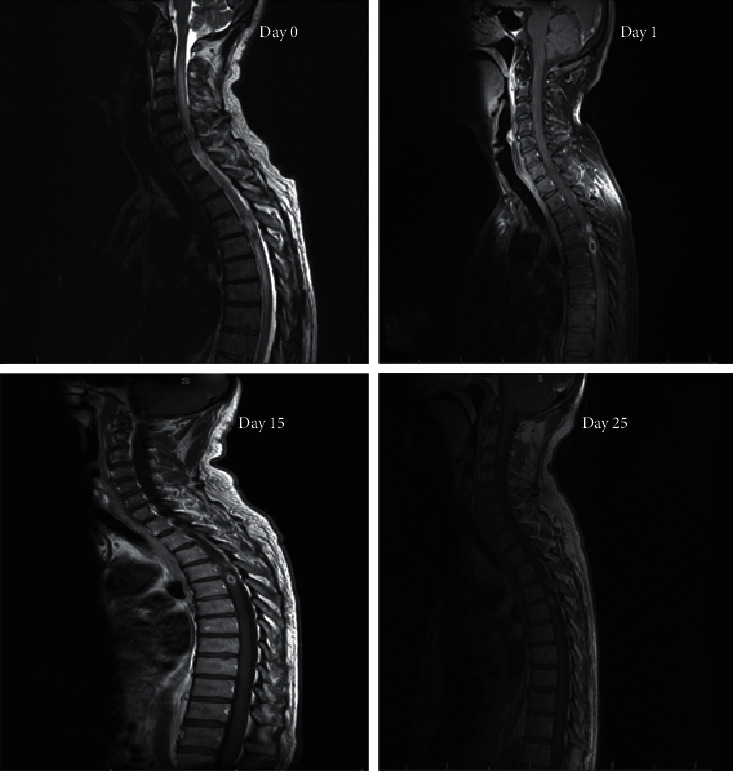
MRI of the spine with gadolinium contrast performed at days 0, 1, 15, and 25. Initial examination revealed a small cavitating mass (7.3 mm) within the left thoracic hemicord at the level of T5 and nodule (4 mm) at T4 with associated marked cord oedema and meningeal thickening which peripherally enhanced on contrast administration. By day 25, the lesion had shrunk to 5.5 mm with marked improvement in cord oedema and meningeal thickening. The superior T4 nodule remained unchanged in appearance.

A 54-year-old gentleman with a history of well-controlled HIV established on Truvada and raltegravir, seizures, migraines, and previously treated syphilis presented to Croydon University Hospital with a 4-week history of progressive left leg weakness, bowel and bladder change, and a 1-week history of dysesthesia (Table 2). He denied any infective symptoms, weight loss, and personal or family history of tuberculosis (TB). He had no recent travel and reported no new sexual partners. Clinical examination revealed a normal cardiorespiratory examination. Neurologically, the patient had brisk reflexes in all limbs with reduced power (MRC 0/5) in the left leg combined with sensory dissociation starting from T5 in keeping with Brown-Sequard syndrome. Magnetic resonance imaging (MRI) of the spine undertaken at this time identified two peripherally enhancing cavitating lesions at T4-T5 with associated meningeal thickening and cord oedema. These images were felt indicative of CNS TB. Following this, anti-TB medication was started, and the patient was transferred to St. George's Hospital for further care. Cerebrospinal fluid (CSF) analysis undertaken revealed highly elevated protein (3.07 g/L) and white cell count (7 × 10^9^/L). CSF cryptococcal antigen (CRAG), TB molecular testing (GeneXpert), microscopy and culture, and viral polymerase chain reaction (PCR) were all negative. Serial sputum acid-fast bacilli (AFB), peripheral blood cultures, and Q-interferon tests were negative. At this point, syphilis serological testing was undertaken; this suggested highly active acute infection (serum EIA positive and rapid plasma reagin (RPR) (1 in 64)). CSF serology showed positive *Treponema pallidum* particle agglutination (TPPA) 10240 and RPR 1 in 2 suggesting active disease. This raised the possibility of neurosyphilis with spinal syphilitic gumma. Due to no conclusive TB diagnosis, concurrent neurosyphilis treatment was initiated by way of 14-day high-dose benzylpenicillin therapy commencing on day 11 of presentation. Repeat MRI of the spine on days 12 and 22 of admission revealed incremental improvement in both spinal cord oedema and the size of the T4-T5 lesions (Figure 1). Following this, positron emission tomography-computed tomography (PET-CT) scan was performed on day 41 which showed no significant areas of increased uptake outside of the spine for potential biopsy. This improvement in the spinal disease was associated with progressive and marked improvements in his neurology, and by day 55, the patient could walk several lengths of parallel bars from being previously bedbound.

## 3. Methods

A literature review was conducted in October 2020 utilising the PubMed database. Searches were performed between years 2011 and 2020. Keywords included “Spinal” AND “Neurosyphilis” OR “Gumma” OR “Granuloma.” From these, 26 case reports were identified for review. All the data including neuroimaging characteristics of confirmed neurosyphilis as well as serum and CSF syphilis serology were analysed. Papers were excluded if there was no confirmed diagnosis of neurosyphilis, results were already reported elsewhere, they did not have sufficient data, or they were not in English. Of these, 16 were used in the final review.

## 4. Results and Discussion

Neurosyphilis is an exceedingly rare clinical manifestation of syphilis infection although with certain treponemal subtypes (type 14 d/f), increased rates may be observed. Neurosyphilis is an underappreciated cause of CNS dysfunction and should be actively considered in the setting of HIV as in our patient. Predictors for CNS involvement in HIV include a serum RPR titre 1 : 32, a peripheral CD4 count <350 cells per cubic millimetre of blood, and the absence of antiretroviral therapy [1].

Typical findings of neurosyphilis include a white cell count >5 cells/*µ*L or >20 cells/*µ*L in the setting of untreated HIV, hyperproteinorrachia (>45 mg/dL), and the presence of oligoclonal bands [5]. In our case, our patient satisfied all these criteria. Key to diagnosing syphilis is blood serology with two-stage testing advised utilising both nontreponemal-specific (e.g., RPR or VDRL) and treponemal-specific (e.g., TPPA) tests. While nonspecific serological tests often decline rapidly after treatment and can be used to measure disease activity, treponemal-specific antibodies remain reactive irrespective of treatment history as demonstrated in the historical syphilis testing of our patient (Table 2). Spinal syphilitic gummas however are rare manifestations of the disease and are considerably rarer than their cerebral counterparts with only 12 cases presented in the literature. The formation of gumma is considered a hypersensitivity reaction secondary to either syphilis reactivation or reinfection and can occur in both immunocompetent and immunocompromised patients. Typically, they stain for glial fibrillary acid protein and neurofilament protein on histology however as in our case biopsy may not always be possible [10]. Given our patient's previous infectious history and treatment, while this may represent reactivation of syphilis given the undetectable viral load at the time of presentation, reinfection is felt more likely. Unlike the other reported cases of spinal syphilitic gumma, this represents the only case occurring in the setting of HIV (Table 3). Therefore, one can surmise that cellular immunity is integral to gumma formation; however, due to the patient's excellent compliance with antiretroviral therapy, this may explain its presence here.

To demonstrate neurological involvement, however, in syphilis, CSF serology is required. In order to assist with the diagnosis of neurosyphilis, one must consider intrathecal syphilis antibody production. Firstly, both treponemal and nontreponemal antibodies are produced in the CSF, although at lower levels than in the serum. Secondly, during infection, greater treponemal antibodies are produced in both CSF and serum compared to their nontreponemal counterparts; however, the former of these can passively diffuse into the CSF resulting in an overall low specificity and a high sensitivity. The opposite, however, is true for nontreponemal antibodies where the specificity is high with passive diffusion playing less of a role, but their sensitivity is lower due to the overall less intrathecal production [8]. Given this, the gold standard investigation is considered as the CSF-VDRL which has a reported sensitivity of 30–70% but a specificity of 100% in the absence of blood contamination. If the CSF-VDRL is negative but symptoms are consitent with neurosyphilis, then CSF treponemal tests should be undertaken. Unlike other major guidelines, the British Association of Sexual Health and HIV (BASHH) guidelines advocate the use of CSF-TPPA in the assessment of patients with suspected neurosyphilis with titres >1 : 320, indicative of neurosyphilis with a sensitivity >98% and specificity of 79% reported. The routine use of these CSF-TPPA titres in the diagnosis of neurosyphilis has, in some centres, led to reduced rates of erroneous diagnosis, and in up to 100% affected patients, CSF-RPR positivity has been described as was seen in our patient's CSF analysis. Considering the average cost of managing inpatient neurosyphilis is ∼£4700, this will likely become the standard investigation of choice in the future, superseding the CSF-VDRL as a first-line investigation [8, 11].

At present, the role of polymerase chain reaction (PCR) in syphilis is confined to sampling of mucocutaneous lesions in primary/secondary disease with a reported sensitivity and specificity of >95%. The role of PCR in CSF testing in neurosyphilis however remains in its infancy. A 2018 systematic review reported a sensitivity of between 40 and 70% and specificity of 60–100%. It is important to note that these results were based on CSF-VDRL positivity which may lead to an underestimation of its efficacy but may, upon refinement, provide the basis for infection of the CNS disease in the future [6]. Future diagnostics may include measuring CNS exosomes. These are secreted nanovesicles which encapsulate both microRNA (miRNA) and messenger RNA (mRNA) and can freely pass across the blood-brain barrier into the CSF. Early studies have shown clear upregulation of miR-570-3P, miR-57-5P, and decreased miR-93-3p in neurosyphilis-positive patients versus negative controls. However, the effect HIV infection may have on the relative production of miRNAs remains unknown as does their role in the pathogenesis of CNS infection [12].

While serology offers laboratory diagnosis, imaging is instrumental to rule out disease mimics, particularly if structural lesions are thought likely as in our case, where the patient presented with Brown-Sequard syndrome and altered bowel and bladder function. Of the few cases that exist, 2/12 presented as Brown-Sequard with paraplegia/tetraplegia found in the remainder with a predilection for thoracic disease noted which may represent increased translocation at this site due to its relatively high blood supply versus other areas of the spinal cord. Unlike cerebral gumma, the literature surrounding radiological characteristics of spinal gumma is limited [13]. However, common themes on MRI imaging include T1-weighted imaging iso/hypointensity with associated hyperintensity on T2-weighted imaging. This is often associated with extensive spinal cord oedema and peripheral lesion enhancement on gadolinium injection. Our case agrees with this description which, alongside positive syphilis serology, made us consider neurosyphilitic gumma as the likely cause of our patient's presentation. A key differential, however, of this radiological appearance is CNS tuberculomas. TB remains a significant global health problem, particularly in HIV patients who are at greater risk of extrapulmonary disease. CNS involvement in TB occurs in ∼10% of patients, and tuberculomas are the second most common manifestation of this disease after TB meningitis; however, despite this, it remains rare [14, 15]. MRI characteristics of the spinal tuberculoma depended on the underlying stage of the disease with four stages recognised, of which noncaseous granuloma with liquefaction is the most common. On MRI, this presents as iso/hypointense on T1-weighted imaging with hyperintense ring-like enhancement on T2WI and contrast administration. Susceptibility weighted imaging (SWI) can be useful in tuberculoma investigation where a complete peripheral hypointense ring is observed [16]. It is due to this commonality on MRI, the relatively higher prevalence of CNS TB versus spinal gumma, that CNS TB was first considered in this patient and a 9-month CNS regime of anti-TB medication commenced. Due to the subsequent changes on the MRI of the spine, it was difficult to differentiate if this represented response to either therapy; as such, both therapies were continued alongside long-term clinical review.

Treatment in neurosyphilis is broadly similar to other stages of syphilis disease with high-dose intramuscular and intravenous (IV) penicillin used. Typically, treatment regimens include 2.4 to 24 million units of IV therapy over a 10–14-day period. Our approach mirrored recent BASH guidelines which suggest 2.4 g benzylpenicillin over 14 days. Commonly, steroids are given during the first 3 days starting 24 hours prior to the initiation of penicillin therapy to prevent Jarisch–Herxheimer reaction [4, 7, 10, 17, 18]. At present, resolution of CSF abnormalities evaluated at 6-month intervals is seen as the best means to determine cure with serological cure considered as a four-fold decline in CSF-TPHA titres within 12 months of therapy [19]. In our case, repeat CSF titres were not taken; however, incremental MRI demonstrated improvement which corresponded with his neurology. While, in the context of cerebral syphilitic gumma, complete resolution can often be seen, spinal gumma is associated with variable recovery, and the neurological sequelae may be permanent; however, long-term outcomes are mostly unknown. Of those with recorded outcomes, three cases showed excellent recovery upon receiving medical therapy, with no superior outcomes noted in those patients who had preceded this lesionectomy or nonpenicillin-based medical therapy [10]. As such, surgical treatment should be considered only in patients with rapidly progressive neurology before systemic penicillin therapy can be administered. Our patient demonstrates partial but significant recovery, and we hope with long-term neurorehabilitation which he is currently receiving, this will continue to improve.

In conclusion, this case represents a rare instance of spinal syphilitic gumma which has diverse imaging and clinical features and should be considered in HIV-infected patients presenting with structural spinal cord lesions and Brown-Sequard syndrome. To diagnose neurosyphilis, a comprehensive clinical history alongside serological testing of blood and CSF is instrumental. Where the diagnosis remains uncertain, a course of high-dose penicillin may offer the best choice with consideration of close mimics including TB through thorough investigation.

## Figures and Tables

**Table 1:** The stages of syphilis infection and their clinical features 5.

Stage of syphilis	Time period	Clinical features
Primary	9–90 days	Chancre
Secondary	6–12 weeks	Alopecia, palmar rash, condyloma lata
Early latent	<1 year	Nil but positive serology
Late latent	≥1 year	Nil but positive serology
Tertiary syphilis	1–20 years	Cardiovascular syphilis Neurosyphilis Gummatous syphilis

**Table 2:** Summary of historical serum syphilis investigations.

Year	EIA total	TPPA	RPR	IgM
2015	—	Neg	Neg	N/A
2018	Detected	1 : 20480	1 : 32	Equivocal
2019	Detected	1 : 20480	1 : 2	Neg
2019	Detected	1 : 20480	1 : 4	Neg
2019	Detected	1 : 20480	1 : 4	Neg
2019	Detected	1 : 20480	1 : 32	Neg
2020	Detected	1 : 20480	1 : 16	Neg

**Table 3:** Summary of all previously recorded cases of spinal gumma neurosyphilis in the literature [3, 11–13].

Age (gender)	Presentation	Radiological features
65 (F)	Tetraplegia	T1WI isotense, T2WI hyperintense, Gd-peripheral enhancement
40 (M)	Paraplegia	T1WI isotense, Gd-ring enhancement
49 (F)	Paraplegia	T1WI isotense, T2WI hyperintense, Gd-homogenous enhancement
47 (F)	Paraplegia	T1WI and T2WI isotense, Gd-peripheral enhancement
25 (F)	Brown-Sequard syndrome	T1WI hypointense, T2WI hyperintense (isotense centre)
48 (F)	Paraplegia	—
51 (M)	Pain, right leg weakness	Gd-peripheral enhancement
51 (M)	Paraplegia	T1WI hypointense, T2WI hyperintense, Gd-ring enhancement
45 (F)	Brown-Sequard syndrome	T1WI slight hyperintensity, T2WI hyperintense, significant
22 (M)	Right leg weakness	Gd-homogenous and ring enhancement
58 (M)	Tetraplegia	T1WI hypointense, T2WI hypointense, Gd-enhancement
46 (M)	Bilateral lower limb sensory loss	Extensive oedema, T2WI hyperintense periphery, hypointense
